# BIAdb: A curated database of benzylisoquinoline alkaloids

**DOI:** 10.1186/1471-2210-10-4

**Published:** 2010-03-05

**Authors:** Deepak Singla, Arun Sharma, Jasjit Kaur, Bharat Panwar, Gajendra PS Raghava

**Affiliations:** 1 Bioinformatics Centre, Institute of Microbial Technology (CSIR), Chandigarh, India

## Abstract

**Background:**

Benzylisoquinoline is the structural backbone of many alkaloids with a wide variety of structures including papaverine, noscapine, codeine, morphine, apomorphine, berberine, protopine and tubocurarine. Many benzylisoquinoline alkaloids have been reported to show therapeutic properties and to act as novel medicines. Thus it is important to collect and compile benzylisoquinoline alkaloids in order to explore their usage in medicine.

**Description:**

We extract information about benzylisoquinoline alkaloids from various sources like PubChem, KEGG, KNApSAcK and manual curation from literature. This information was processed and compiled in order to create a comprehensive database of benzylisoquinoline alkaloids, called BIAdb. The current version of BIAdb contains information about 846 unique benzylisoquinoline alkaloids, with multiple entries in term of source, function leads to total number of 2504 records. One of the major features of this database is that it provides data about 627 different plant species as a source of benzylisoquinoline and 114 different types of function performed by these compounds. A large number of online tools have been integrated, which facilitate user in exploring full potential of BIAdb. In order to provide additional information, we give external links to other resources/databases. One of the important features of this database is that it is tightly integrated with Drugpedia, which allows managing data in fixed/flexible format.

**Conclusions:**

A database of benzylisoquinoline compounds has been created, which provides comprehensive information about benzylisoquinoline alkaloids. This database will be very useful for those who are working in the field of drug discovery based on natural products. This database will also serve researchers working in the field of synthetic biology, as developing medicinally important alkaloids using synthetic process are one of important challenges. This database is available from http://crdd.osdd.net/raghava/biadb/.

## Background

Alkaloids are naturally occurring secondary metabolites, low molecular weight, and nitrogen containing compounds that are found in more than 20% of plant species [1]. A plant contains more than 0.01% of alkaloids is called alkaloid plants [2-4]. They show pharmacological effects and are being commonly used as medicines (e.g., analgesic as morphine). Alkaloids are biologically significant and can act as stimulators, inhibitors and growth terminators [5,6]. They also have anti-microbial and anti-parasitic properties [7-14]. Alkaloids can alter DNA, selectively deform cells and cause locoism. Nowadays, non-natural alkaloids are growing rapidly as a result of bioorganic and stereochemistry research. Pharmacological research and the drug industry rapidly advance and promote the most promising new molecules for possible production applications [15].

Alkaloids can be classified on the basis of biological activities, chemical structure and biosynthetic pathways. Following are few of major classes of alkaloids; (i) Pyrrolidine: is an organic compound with the molecular formula C_4_H_9_N; (ii) Quinoline: is a heterocyclic aromatic organic compound with formula C9H7N; (iii) Benzylisoquinoline: is a heterocyclic aromatic organic compound & structural isomer of quinoline; (iv) Indole: is an aromatic heterocyclic organic compound (v) Terpenoid: a diverse class of naturally occurring organic chemicals. The benzylisoquinoline alkaloid (BIA) is a diverse category of alkaloids including berberine, morphine, sanguinarine, hydrastine and many more. Alkaloids belonging to this class are found to be pharmacologically active and show potential therapeutic properties. Recent studies suggested that these alkaloids could be considered as novel medicines [2]. For example, the magnoflorine has been reported to protect HDL during oxidant stress [16-18]. A recent report stated that anti-microbial agent berberine had cholesterol-lowering activity [19]. Tetrandrine (TET) is a bis-benzylisoquinoline alkaloid, which is identified as an active ingredient in Radix Stephanae tetrandrae (a Chinese medicinal herb). It has been used traditionally for the treatment of congestive circulatory disorder and inflammatory diseases [20].

Best of authors' knowledge there is no databases of BIA molecules, which are very important from medicine point of view. In order to facilitate researchers working in the field of drug discovery/design, we made a systematic attempt in this study to collect and compile BIA molecules. This database is user-friendly and equipped with powerful computational tools like Jmol [21]. BIAdb also has provision for searching the chemical compounds against this database on the basis of structural similarity.

## Construction and content

### BIAdb Resources

BIAdb contains; (i) comprehensive information about BIA's, (ii) tools for 2-dimensional and 3-dimensional structure visualization, (iii) hyperlinks to related databases, and (iv) tools for searching structurally similar molecules. BIAdb contains manually curated data as well as from different sources like public databases [22-25]. Total 846 unique BIAs have been incorporated into the database till date. The statistics of the data has been shown in Table 1.

**Table 1:** Number of unique BIA molecules from different data sources

DATA SOURCE	NUMBER OF ENTRIES
**KEGG (COMPOUNDS & DRUGS)**	196
**Comparative Toxicogenomics Database (CTD)**	145
**PubChem**	171
**Other Sources (literature search)**	334
**TOTAL**	846

### Data Structure

The BIAdb database contains the following fields for each benzylisoquinoline alkaloid entry; (1) name; (2) PubChemID; 3) KEGG ID; (4) source; (5) type; (6) function; (7) molecular weight; (8)exact mass; (9) molecular formula; (10) XLogP; (11) Topological Polar Surface Area (TPSA); (12) IUPAC name; (13) H-bond donors; (14) H-bond acceptors; (15) number of rotatable bonds; (16) canonical smile; (17) isomeric smile; (18) structure (sdf, mol, pdb) file;(19) pubmed web-link; and (20) link to Drugpedia.

### Organization of Data

The whole data of BIA's compounds was organized in four different ways, these are physio-chemical properties, sources, functions and clustering of whole data was done using standalone version of LibraryMCS software. The organization of data on the basis of physio-chemical properties can be retrieve through the browse option provided in the web server. The data has been organized according to the different ranges of the molecular weight, XLogP, TPSA of BIA's.

The whole data was clustered using the standalone LibraryMCS v0.7 from ChemAxon. LibraryMCS clusters a set of chemical structures on a structural basis. Structures that share a common substructure are clustered together. The clustering program identifies the common substructure, and it is always the largest one among all substructures found in the structure set. Such substructure is called the Maximum Common Substructure (MCS). The clustering technique applied in LibraryMCS is hierarchical, that is, clusters of input structures are grouped into second level clusters, and then these second level clusters are grouped again and so on, until a termination condition is reached.

The matching parameters for clustering were atom type, bond type, charge, hybridization and isotopes, minimum cluster size. Performance of LibraryMCS software depends upon various factors like average structure size, diversity, minimal required MCS size and atom/bond constraints.

**Figure 1:**
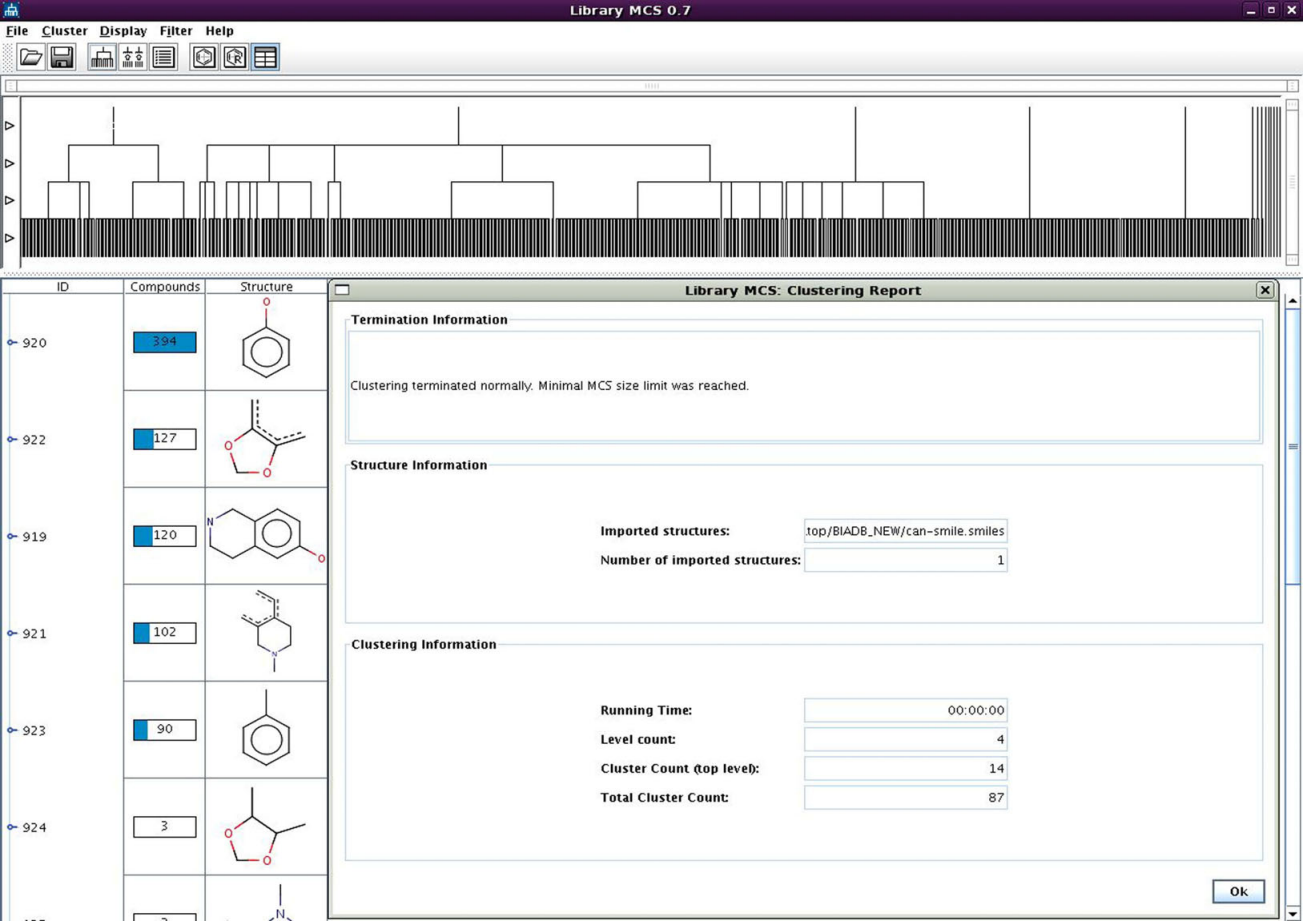
**LibraryMCS clustering report**. Figure shows a clustering result of BIA's Compounds, Top level show a cluster of compounds at four different levels, left side shows the cluster id, number of compounds in a cluster and the base structure use for clustering. Right side shows the output in the form of input file, time taken for clustering, cluster level, top level cluster and total number of cluster.

The file containing structural information of all 846 compounds was given as input to the software using minimum cluster size 7 under normal speed. LibraryMCS generated 14 top-level clusters and total of 87 clusters with four levels of hierarchy. Figure 1. shows the LibraryMCS clustering report.

The data was grouped according to the top-level clusters. The groups were named Group 1 to Group 14 each containing different number of input compounds. Table 2 Indicates the total number of compounds in each of 14 top level clustered groups obtained using LibraryMCS software.

**Table 2:** Number of compounds in 14 Top level clusters obtained from LibraryMCS

GROUPS	ID	Number of Compounds in each cluster
**Group 1**	920	394
**Group 2**	922	127
**Group 3**	919	120
**Group 4**	921	102
**Group 5**	923	90
**Group 6**	924	3
**Group 7**	925	2
**Group 8**	926	2
**Group 9**	927	1
**Group 10**	928	1
**Group 11**	929	1
**Group 12**	930	1
**Group 13**	931	1
**Group 14**	932	1

## Utility

### Web Tools

Apart from the data collected about benzylisoquinoline alkaloids, a variety of tools has been implemented within the database to enhance the scope of database. Following are the main web tools integrated in BIAdb.

#### Keyword Search and Advanced search

A simple text search tool is provided for searching on selected fields of database. The advance search option has been incorporated separately for intensive search. Using this option, users can search BIA's compounds within a range of value of physiochemical properties and retrieve corresponding results.

The browsing tool provides the options to the user to select particular range of compounds on the basis of their physio-chemical properties like molecular weight, XLogP and TPSA.

#### Structure visualization and similarity Search

**Figure 2:**
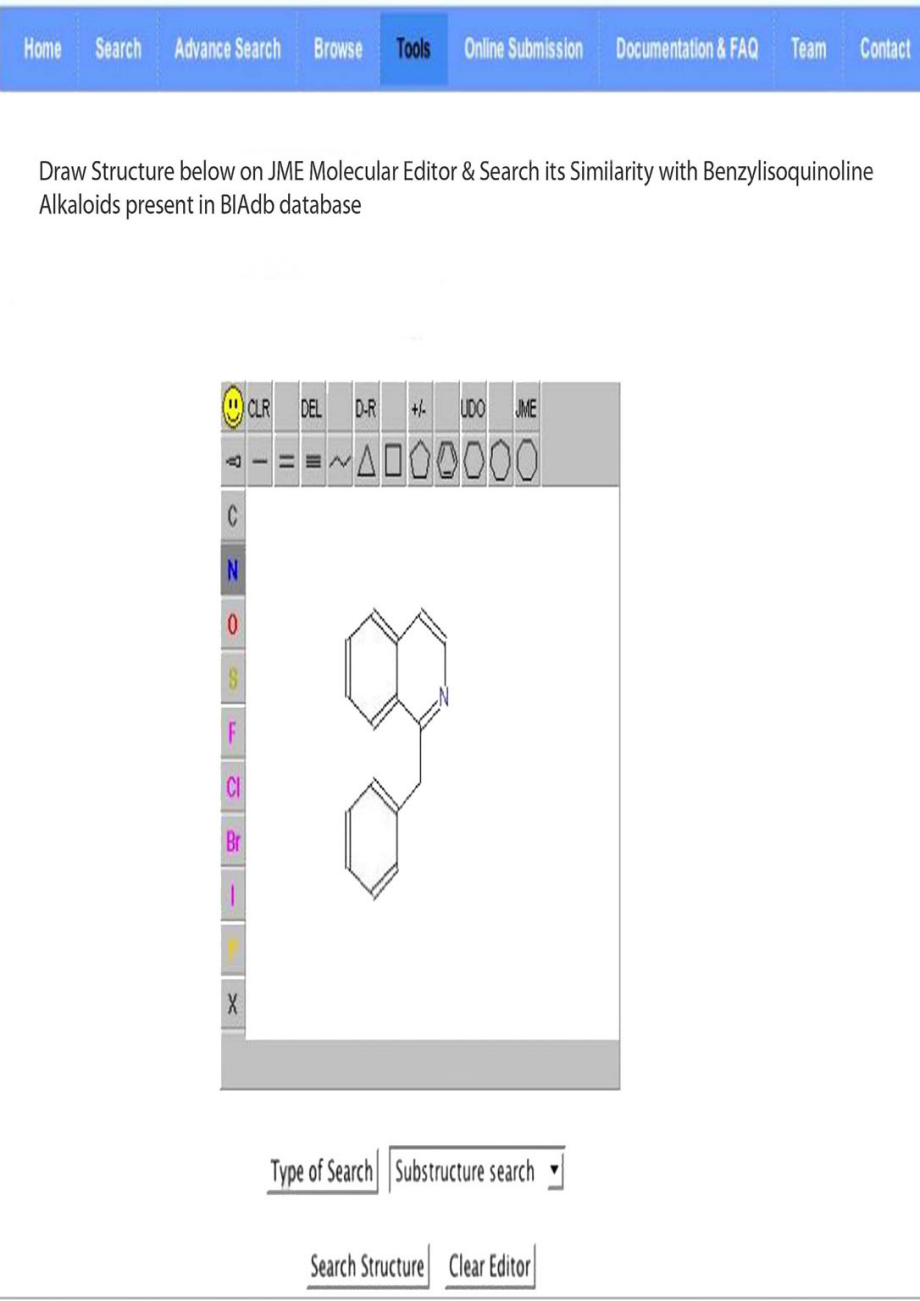
**Structure search tool in BIAdb**. An example of a structure drawn in JME and search against BIAdb by selecting substructure search, using JC search tool.

**Figure 3:**
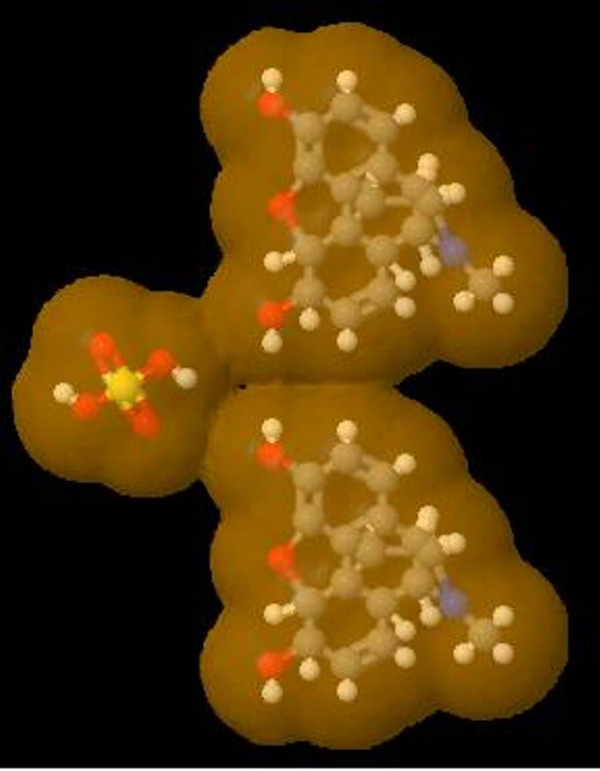
**Structure of Morphine visualized by Jmol**. Structure of the morphine visualized by Jmol, in ball-sticks model representing the van der Waals suface, showing different labels of atoms; white balls are hydrogen atom, red oxygen, yellow sulphur, blue nitrogen and gray are carbon atoms.

A simple Java based JME (Java Molecular Editor) [26] tool, has been provided to draw structure to be searched in the database using JC search tool [27]. The tool provides substructure, exact, super structure, perfect and simple searching options. Figure 2 depict the structure search tool provided in the database. BIAdb facilitates the users to display 2-D and 3-D structure of molecules using Jmol. User is provided with many options to visualize the structure in many different ways. Figure 3 represents the van der Waals surface representation of morphine.

#### Online submission tool

BIAdb also has the facility to add more entries related to benzylisoquinoline alkaloids by the users. The user can add new compound information with in specified fields (the fields with star are mandatory) and entries will be then added to the database after proper validation.

#### Drugpedia

**Figure 4:**
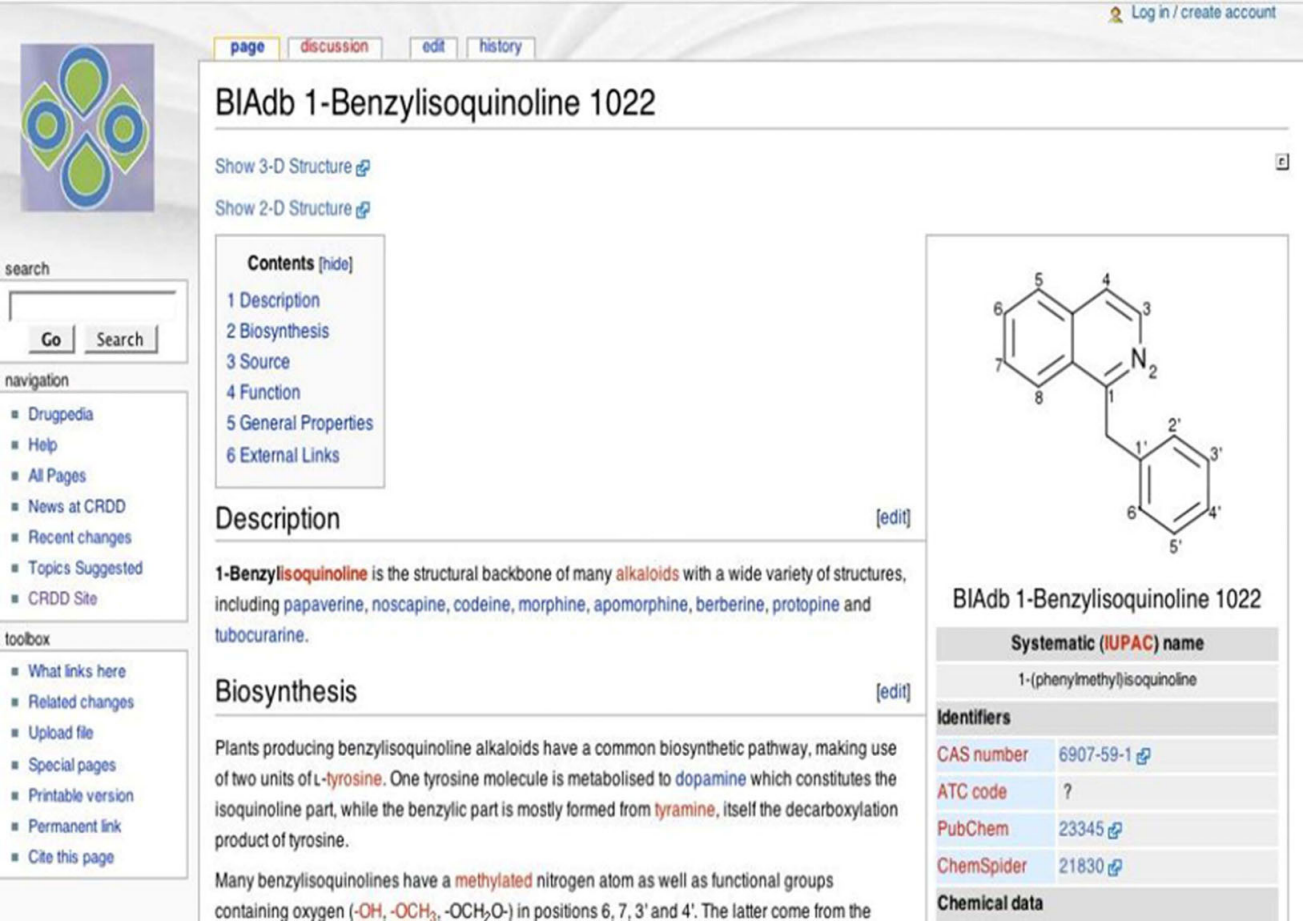
**Drugpedia page of BIAdb 1-Benzylisoquinoline 1022**. Figure shows a Drugpedia page for BIAdb 1-Benzylisoquinoline 1022. Top right corner contains link for new account creation and login. For searching a search option is provided in the middle left. To edit, discuss, or see history of a particular page, links are embedded on top left of the page

BIAdb provides a Drugpedia [28] link corresponding to each single entry. Data hosted in BIAdb platform follow a definite pattern. Therefore, to provide more flexibility to different kinds of data a Drugpedia link would be beneficial, where user can update or add any relevant information. The important and relevant information from Drugpedia page may be included in the main database frame after processing and validation, during the updating of BIAdb. Figure 4 illustrates a typical Drugpedia page for BIAdb 1-Benzylisoquinoline 1022.

### Data Flow

**Figure 5:**
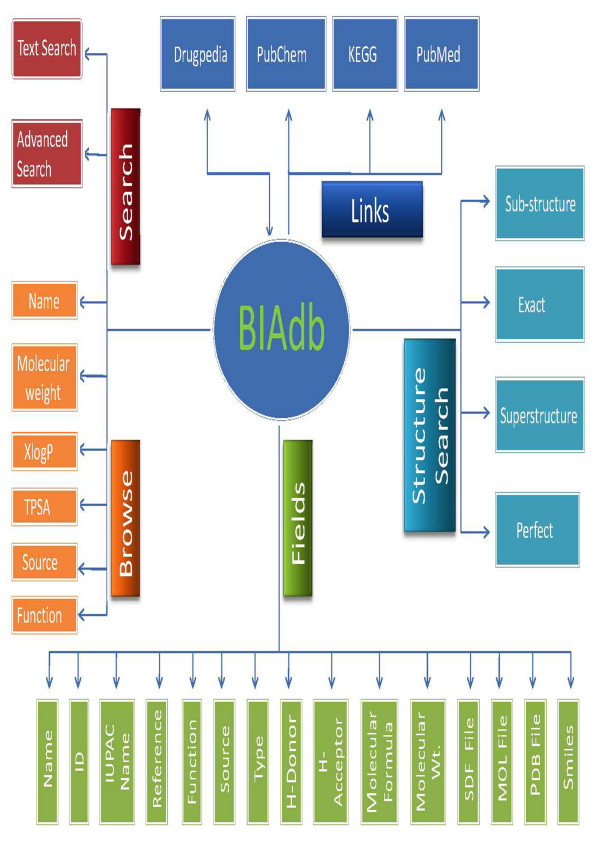
**Knowledge flow in BIAdb**. Data flow for BIA's have been shown in different color schemes; red colored fields show search tool, orange color shows browse tools, blue color shows link to other databases, green color depict the data fields and sky blue color structure search tool.

Text search option operates on data type like BIA's name e.g. "morphine". The advance search page provides variety of options to query the database. User can use data types like name of the compound, molecular weight, XLogP, Polar Surface Area ranges. Query search term "morphine" retrieves number of entries related to "morphine". Users can click on the BIA ID of particular entry to retrieve the detailed information about the "morphine". The two-dimensional as well as three-dimensional structures of morphine can be visualized by clicking 2-D & 3-D structure buttons in the 2-D & 3-D structure field. In addition to this user can also download the structure coordinates file in different format like SDF, MOL, and PDB by a clicking on their respective field. Figure 5 depicts the knowledge flow in BIAdb.

### Web Interface and Application

LAMP (Linux-Apache-Mysql-PHP), an open source software has been used to create the database. PHP, HTML and CSS technologies have been used to build the dynamic web interface. MySQL, a relational database management system (RDBMS), works at the backend. Server-side scripting makes use of PHP. The whole software system runs on IBM SAS x3800 machine under Red Hat Enterprise Linux 5 environment using Apache httpd server. PHP and MySQL combination is quite efficient and powerful for database management.

## Discussion

BIAdb is a comprehensive information portal of benzylisoquinoline alkaloids. User can search important information regarding the BIA's and can also view the structures of the compounds. This database will definitely be of great use for researchers. BIAdb is developed keeping in mind the general user requirement. Thus searching and exploring the database is easy and convenient. Further, this information can be used to design analogs of BIA's, which can act as potential drug candidates.

## Conclusion

BIAdb is a webserver of an important class of alkaloids called benzylisoquinoliones which facilitate users to do extensive search. This database encapsulates important information about BIA's compounds regarding their source and medicinal values. The structure search facility will be helpful for users in finding compounds similar to BIA's. The online submission facility will be helpful for expanding this database. This resource will be helpful for those working in the field of drug designing to explore their role as potential drug molecules.

## Limitations and future prospects

Our major limitation in developing the database is that the information about benzylisoquinoline alkaloids is very less and too scattered. Lot of literature search is required to further expand the database. In order to maintain the database dynamically without losing any information about BIA's, we make the multiple records in term of different source and function performed by a compounds. In near future, we are hopeful of expanding the database both qualitatively as well as quantitatively to cover the synthesis pathways of these BIA's and to include the synthetic parts available to shorten the synthesis process and to increase the yield. This database will be updated manually as soon as enough data will be available.

## Availability and requirements

BIAdb is available at http://crdd.osdd.net/raghava/biadb/ and its mirror site http://www.imtech.res.in/raghava/biadb. To access BIAdb World Wide Web is a pre-requisite. To access all features of BIAdb to its optimum level, JavaScript and Java Runtime Environment (JRE) plugin must be enabled.

## List of abbreviations used

BIA: Benzylisoquinoline Alkaloids; CTD: Comparative Toxicogenomics Database; PHP: Hypertext Preprocessor language; HTML: Hypertext Markup Language; CSS: Cascading Style Sheet.

## Authors' contributions

JKB, BP and DS collected and compiled the data from literature and public databases and made the structure of the database. In second phase, DS further refined the database and add additional information like source, function. AS work extensively on development and implementation of web tools and web interface. DS, JKB and BP drafted the manuscript. GPSR conceived the project, coordinated it and refined the manuscript. This manuscript has been seen and approved by all the authors.
